# Comparison of Swallowing Act Videofluoroscopy after Open and Laser Partial Supraglottic Laryngectomy

**Published:** 2018-11

**Authors:** Mario Bilic, Lana Kovac-Bilic, Selma Hodzic-Redzic, Drago Prgomet

**Affiliations:** 1 *Department of Otorhinolaryngology, Head and Neck Surgery, Clinical Hospital Center Zagreb, Zagreb, Croatia.*

**Keywords:** Deglutition, Deglutition disorders, Endoscopy, Gas, Laryngeal neoplasms, Laryngectomy, Lasers

## Abstract

**Introduction:**

The aim of this study was to compare the functional outcomes of swallowing act detected by videofluoroscopy of two different techniques in the treatment of laryngeal carcinoma.

**Materials and Methods:**

This study was conducted on 41 patients undergoing two supraglottic laryngectomy techniques. The research population was assigned into two groups of open and laser supraglottic laryngectomy, including 21 and 20 patients, respectively.

**Results:**

Food residue was present in most of the patients in the open laryngectomy group. Aspiration of the liquid and solid contrasts was observed in 16 and 4 patients, respectively. In the laser laryngectomy group undergoing a partial supraglottic laryngectomy via carbon dioxide (CO_2_) laser, aspiration was recorded in only six patients. There was a statistically significant difference between these two groups regarding the presence of aspiration as a marker of a bad functional outcome.

**Conclusion:**

Techniques that include the endoscopic removal of the tumor via CO_2_ laser result in good oncologic and functional outcomes, along with reduced postoperative morbidity and mortality.

## Introduction

The treatment of supraglottic laryngeal carcinoma traditionally involves surgery and radiotherapy or both. Improvement in surgical techniques has led to fundamental changes in the treatment of patients suffering from supraglottic laryngeal carcinoma. The purpose of the new approaches is to preserve laryngeal function; however, the fundamental principles of oncology should be considered to decide about the extent of the surgery. 

In open partial supraglottic laryngectomies, the major postoperative consideration is an aspiration in the latent or clinical manifestation, which is caused by the resection of protective barriers that normally exist, such as epiglottis, aryepiglottic, and false vocal folds (1). Besides, open supraglottic laryngectomy demands the use of tracheostomy and feeding tubes during the postoperative period (2). With the wide acceptance of microlaryngeal surgical techniques, CO_2_ laser has gained importance in conservative surgical approaches. Some authors reported on microlaryngealtransoral resection via CO_2_ laser in the management of early supraglottic tumors with the emphasis on faster recovery of the swallowing function and usually exclusion of the need for additional tracheostomy (3,4).

Furthermore, both procedures (i.e., open and endoscopy techniques)are still widely accepted. 

The aim of the present study was to compare functional results after open supraglottic laryngectomy and supraglottic laryngectomy via CO_2_ laser using the videofluoroscopy of the swallowing act. 

## Materials and Methods

This prospective study was conducted on 41 patients operated at the Clinical Hospital Center Zagreb, Croatia. The inclusion criterion was the lack of any surgeries, except the preoperative tumor biopsy, which is an obligatory step in tumor diagnosis. In addition, none of these patients went through preoperative radiotherapy. On the other hand, the exclusion criteria were history of previous neck surgeries and preoperative treatment with radiotherapy. The operation extent was determined by the tumor stage. 

The patients were randomly divided into two groups of open and laser techniques. The open group consisted of 21 patients (i.e.,male:20, female:1) aged 46-68 years that went through open partial supraglottic laryngectomy. Supraglottic laryngectomy included the detachment of the laryngeal framework, resection of the epiglottis, aryepiglottic, and ventricular folds. The laser group included 20 patients (i.e., male: 18, female: 2) aged 47-69 years that underwent partial supraglottic laryngectomy by CO_2_ laser. Standard endoscopic supraglottic laryngectomy (SGL), including the removal of the epiglottis, pre-epiglottic space, and at least one aryepiglottic and false vocal fold, with the preservation of thyroid cartilage was performed on 12 patients. Furthermore, five patients were subjected to epiglottectomy. Extended endoscopic supraglottic laryngectomy (ESGL), entailing the resection of the vallecula and part of the base of the tongue, was performed on three patients. A nasogastric feeding tube was intraoperatively placed in all patients and removed approximately on the seventh postoperative day. Videofluoroscopy of the swallowing act was carried out in posteroanterior and lateral projections (25 frames per sec) at least 6 months after the surgery. Barium, functioning as contrast media, was used as liquid bolus; in addition, bread imbibed by barium was employed as a solid bolus. None of the patients received any kind of additional therapy (e.g., radio-or chemotherapy) during the surgery until the test was performed. 

***Statistical analysis***

The data were analyzed using Medcalc Statistical Software, version 15.8 (MedCalc Software bvba, Ostend, Belgium). The Differences between two groups of the patients were calculated by means of the Chi-square test. P-value less than 0.01 was considered statistically significant.

## Results

**Fig 1:**
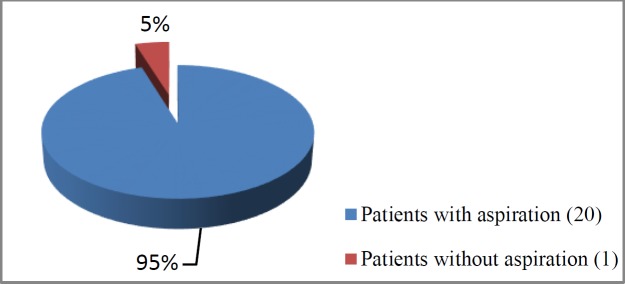
Aspiration among patients that underwent open partial supraglottic laryngectomy

**Fig 2:**
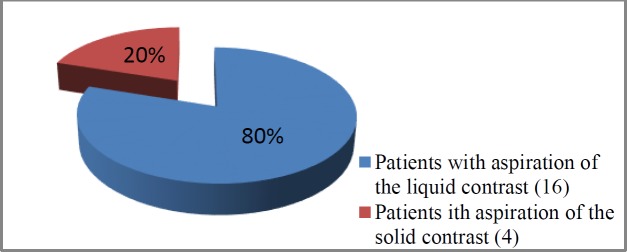
Aspiration of the solid and liquid contrast among patients that underwent open partial supraglottic laryngectomy

**Fig 3:**
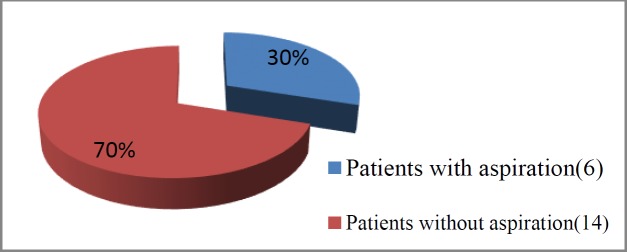
Aspiration among patients that underwent partial supraglottic laryngectomy by CO_2 _laser

Out of the 41 patients included in the study, 21 and 20 cases aged 46-68 and 47-69 years underwent open and CO_2_ laser partial supraglottic laryngectomy, respectively. The aspiration of the liquid and solid contrast was observed in 16 and 4 patients in the open laryngectomy group, respectively (Fig1,2). Regarding the laser group, aspiration was observed in six patients, only four of whom had clinical symptomatology (Fig.3). 

Intra-deglutitive (during swallowing) aspiration and both intra- and post-deglutitive (after swallowing) aspiration were reported in one patient, whereas four patients had post-deglutitive aspiration. There were no signs of aspiration after epiglottectomy; however, two of three patients had aspiration after ESGL. No swallowing or other structural abnormalities were detected in this group of patients. (Table.1)

**Table 1:** presents the difference between two groups of patients treated with two different methods

**Patient group**	**Patients with aspiration**	**Patients without aspiration**	**P-value**
Patients undergoing open partial supraglottic laryngectomy	20	1	P<0.01
Patients undergoing partial supraglottic laryngectomy via CO_2 _laser	6	14	

Chi square test 18.7919; P 0.000015; statistically significant for P<0.01

## Discussion

According to the results, food residues were present in 20 patients in the open laryngectomy group. The aspiration of liquid and solid contrasts was observed in 16 and 4 patients, respectively. In the majority of the cases, food remnants were located in the glossopharyngeal recess. All patients with solid contrast aspiration and two cases with liquid contrast aspiration demanded total laryngectomy, which was performed between 1-3 months after this test. Other patients with aspiration were successfully rehabilitated and did not need any additional surgical procedures. 

In the laser group, six patients had aspiration, one of whom demonstrated both intra- and post-deglutitive aspirations. This case was managed with a permanent nasogastric feeding tube, followed by total laryngectomy performed nine months after the first procedure. Other patients with aspiration were rehabilitated and did not request any kind of additional procedures. There was a statistically significant difference observed between these two groups in terms of functional outcome and need for an additional procedures respectively.

Peretti et al. revealed that patients treated with endoscopic supraglottic laryngectomy had a significantly better swallowing than those managed with the open technique. Furthermore, these patients also spent less time in the hospital (5). Sasaki et al. demonstrated the role of the glottic closure reflex in the patients treated with open endoscopic supraglottic laryngectomy and those managed with laser endoscopic supraglottic laryngectomy. In the mentioned study, the reflex was untouched in the endoscopy group, while the lack of the glottic closure reflex was observed in seven out of eight patients in the laser group (6). 

Perez Delgado et al reported 53 patients treated with supraglottic laryngectomy via CO_2_ laser during 6 years. The patients were followed up for more than 2 years. The results of the mentioned study demonstrated that one patient received total laryngectomy due to the impaired swallowing, while others did not demand any additional procedures (7). Moreover, in some studies, such as the one performed by Chiesa Estomba et al., there were no significant differences between these two groups in functional outcome, and in terms of demanding any additional procedures respectively (8).

Swallowing dysfunction is a common complication after laryngeal surgery that is well-visualized by videofluoroscopy. In the current study, the residues of liquid and solid contrasts were observed in most of the patients after open supraglottic laryngectomy. Furthermore, the aspiration of liquid contrast was noticed in the majority of our participants. According to Kreuzer and Bumber, the aspiration can be due to defective laryngeal closure or pharyngeal pooling (9,10).

In addition, Alicandri-Ciufelli et al. studied more factors that can influence the functional outcome, such as the type of partial laryngectomy, presence of both arytenoids or one arytenoid, and effect of the postoperative radiotherapy. In this regard, only postoperative radiotherapy had a significant influence on the swallowing function (11). Breunig et al. reported that the functional outcome of open supraglottic partial laryngectomy is determined by the tumor extension on the base of the tongue (12). Removal of the malignant tumor includes the resection of extrinsic infrahyoid muscles and soft tissue that are part of the laryngeal framework. The continuity of the aerodigestive tract is achieved through various reconstructive techniques. Relearning the swallowing is reduced in patients after the resection of the epiglottis or arytenoid cartilage (13).

On the other hand, malignant tumor removal via CO_2_ laser allows the resection of the tumor, along with the preservation of uninvolved extrinsic muscles, soft tissues, or laryngeal nerves. These advantages result in a reduced need for permanent tracheostomy and feeding tube, as well as faster rehabilitation. Transoral robotic surgery (TORS) is a new interesting surgical alternative for the two described techniques. The TORS, as well as radiotherapy or radiochemotherapy, represents one of the five main treatment options for the supraglottic laryngeal carcinoma. These options can be used together or separately (8). 

In 2006, TORS was described for the first time. In 2007, Solares et al. performed and wrote up the first supraglottic partial laryngectomy with TORS on a dog model, then a cadaver, and later a female human being with laryngeal cancer (14). Afterwards, Weinstein and other researchers described the advantages of this technique, such as better visualization of the operative field due to higher definition of images, improved surgical skills resulting from robotic arms, and reduction of physiological tremor, which lead to better comfort for the surgeon and less time for the surgery (8,14,15). 

Only a few centers in the world are currently familiar with TORS technique; therefore, the real advantages and disadvantages of this approach will be revealed in the future. Consequently, until the achievement of this end, CO_2_ laser supraglottic laryngectomy would remain as the treatment of choice.

## Conclusion

Patients suffering from dysphagia after laryngeal surgery may have functional and structural abnormalities depending on the surgical technique. Functional outcome of these operations is primarily measured by the extent and type of postoperative aspiration, which was significantly lower in the CO_2_ laser group than in the open group. Our results demonstrated that total laryngectomy more often occurred in patients with supraglottic laryngectomy. Techniques that include endoscopic tumor removal by CO_2_ laser permit good oncologic outcomes, along with reduced postoperative morbidity and mortality, social costs, and swallowing problems. 
